# Strategies used by nurses regarding underreporting of rural work accidents due to pesticide use

**DOI:** 10.1590/0034-7167-2023-0384

**Published:** 2024-06-14

**Authors:** Dionatan Carmini de Brito, Daiani Modernel Xavier, Alberto de Oliveira Redü, Luciano Garcia Lourenção, Franciele Gomes Soares, Giovana Calcagno Gomes, Laura Fontoura Perin

**Affiliations:** I Universidade Federal do Rio Grande. Rio Grande, Rio Grande do Sul, Brazil

**Keywords:** Underregistration, Occupational Health, Occupational Accidents, Nursing, Rural Areas, Omisiones de Registro, Salud Laboral, Accidentes de Trabajo, Enfermería, Medio Rural

## Abstract

**Objective:**

To learn the strategies used regarding underreporting of pesticide use in rural areas.

**Methods:**

A qualitative study was carried out in eight primary healthcare units in rural areas and two emergency care units in a municipality in southern Brazil. Data collection took place in 2023 through interviews. Twenty professional nurses participated. The data was submitted to content analysis.

**Results:**

The strategies identified were lifelong and continuing education for the professionals who carry out the notification, active search and training of workers who deal directly with this type of substance, computerizing the notification by filling in the forms online, and carrying out research on the subject.

**Final considerations:**

Nurses play an important role in reporting occupational accidents caused by the use of pesticides, improving occupational safety in rural areas.

## RESUMEN

**Objetivo:**

Conocer las estrategias utilizadas para el subregistro del uso de plaguicidas en áreas rurales.

**Métodos:**

Estudio cualitativo realizado en ocho unidades de atención primaria en áreas rurales y dos unidades de atención de emergencia en un municipio del sur de Brasil. La recopilación de datos se realizó en 2023 mediante entrevistas. Participaron 20 profesionales de enfermería. Los datos fueron sometidos a análisis de contenido.

**Resultados:**

Las estrategias identificadas fueron la educación continua y permanente de los profesionales que realizan la notificación, la búsqueda activa y la capacitación de los trabajadores que tratan directamente con este tipo de sustancia, la informatización de la notificación mediante el llenado de los formularios en línea y la realización de investigaciones sobre el tema.

**Consideraciones finales:**

Los enfermeros desempeñan un papel importante en la notificación de accidentes laborales causados por el uso de plaguicidas, mejorando la seguridad laboral en las zonas rurales.

## INTRODUCTION

The agrarian landscape in Brazil has undergone major changes, which are directly linked to the project to modernize agriculture, especially with the restructuring of production in the countryside. As a result, agricultural policies have been created to modernize certain regions of Brazil, so that monoculture production can meet the needs of agro-industries and the demands of the foreign market, leading to the mass use of pesticides^(1)^.

Pesticides, also known as agrochemicals, agricultural defensives, or plant protection products, have positive aspects related to the agricultural work process, making it possible to protect crops and plantations against pests, as well as making these crops viable on a large scale. However, if mishandled, they have negative consequences for the health of the exposed population^(2)^. The International Labor Organization (ILO) points out that more than 70,000 acute and chronic poisonings a year occur as a result of pesticide use, often leading to death in developed countries, where more than seven million cases are reported, and in developing countries such as Brazil, pesticide consumption in the agricultural sector has increased significantly^(3)^.

According to the ILO, around 70,000 workers in developing countries die from acute and/or chronic exogenous poisoning caused by the use of pesticides, while another seven million people suffer from non-fatal illnesses related to this practice. Pesticide poisoning can occur through contact with the digestive tract, respiratory tract, skin and/or eyes and can be acute, subacute, or chronic. Among the groups most affected are farmers and ranchers exposed to this type of substance during the preparation of the products and through direct use, without the use of personal protective equipment (PPE), and during the use of packaging after handling the products^(4)^.

The most common form of contamination is direct exposure through the environment, affecting a large part of the population. The main health effects can be acute, which is when symptoms appear quickly, and can include dehydration, skin irritation, allergies, burning nose and mouth, runny nose, cough, chest pain, difficulty breathing, sore throat, stomach pain, nausea, vomiting and diarrhea. Other symptoms considered nonspecific may appear, such as headache, weakness, abnormal sweating, tremors, and irritability^(5)^.

Some groups of pesticides cause cancer in individuals who come into frequent contact with them, making it necessary to take precautions when handling them^(6)^. Exogenous poisoning due to the use of pesticides is considered a public health problem by the Ministry of Health and the World Health Organization (WHO), and is aggravated by underreporting, which causes an information deficit in the Brazilian system. In this sense, for every record of pesticide poisoning made, there are another 50 that go unreported^(7)^.

With the Ministry of Agriculture’s approval for the release of new pesticides, the increase in new records of worker poisoning has indicated an accelerated continuation of this trend. According to the Notifiable Diseases Information System (SINAN), between 2007 and 2017, there were 41,600 cases of poisoning resulting from incorrect or inadequate handling of pesticides, mainly in rural areas^(8)^.

This highlights the importance of reporting these accidents at work. In Brazil, specific regulatory norms (NR, as per its acronym in Portuguese) were created, a priori NR 6, to guarantee prevention against accidents of this nature, and the use of PPE by workers handling these products became more closely monitored^(9)^.

As a result, when an occupational accident occurs, it is important to fill in the compulsory notification form, which is often the responsibility of the nurse. This occupational health information is intended to be used in the community to monitor cases and take measures to promote, protect, and restore health in the workplace^(10)^.

However, underreporting is still a reality in the workplace and it is up to institutions, through their committees, to encourage a process of reflection on its importance. It should be noted that the public health network reports more accidents than the private networks, which serves both to prevent and control these events, protecting the health and safety of workers^(10)^.

As a result, due to the imprecise symptoms reported by farmers and the low demand for health services, exogenous poisoning caused by the use of pesticides is underreported. In small municipalities, misinformation becomes an aggravating factor and, in this sense, seeking strategies through the perspective of health professionals, especially nurses, that contribute to information for health promotion and prevention of harm to the exposed population, becomes a common objective faced by health services^(11)^.

Health professionals should be trained to recognize risk situations and companies should take measures to help achieve these goals, seeking to reduce the underreporting of accidents at work. Although Brazil has one of the best Health Information Systems (SIS), which is capable of recording injuries and illnesses that have occurred in the national territory as a result of problems caused by pesticides, depending on the degree of exposure to health, underreporting often occurs. It is difficult for professionals to determine the causal relationship of pesticide poisoning, the observations of which are important for supporting interdisciplinary actions^(12)^. In this context, it is important to create strategies to prevent the underreporting of occupational accidents caused by pesticide poisoning. Thus, this study had the following guiding question: What strategies do nurses use regarding the underreporting of rural work accidents caused by the use of pesticides?

## OBJECTIVE

To learn the strategies used by nurses regarding the underreporting of rural work accidents caused by the use of pesticides.

## METHODS

### Ethical aspects

The study followed the National Research Ethics Committee/ Ministry of Health (CONEP/MS) Resolution 466/2012 on research with humans+. The nature, objectives, and methods of the research were explained to the participants. Those who agreed to participate signed a free and informed consent form in two copies, one copy remaining with the participant and the other with the researcher.

### Study type

This is a descriptive, exploratory study of a qualitative nature. In order to qualify scientific production on the subject, we adopted the guidelines of the Consolidated Criteria for Reporting Qualitative Research (COREQ)^(14)^.

### Study setting

The setting for this study consisted of eight family health strategy (FHS) units located in rural areas and two emergency care units. The rural units are intended for primary care, but are unavailable on weekends and public holidays. On the other hand, the emergency care units are seen as primary care units, when the rural units are unavailable, and as secondary care units to provide the necessary support for continuity of care, when this begins at the FHS. The units mentioned above are located in a municipality in the southern region of Rio Grande do Sul, Brazil, and provide comprehensive care to the population in accordance with the guidelines of the Brazilian Unified Health System (SUS).

### Data source

Twenty nurses who provided care participated. The requirements for inclusion were: being a nurse duly certified by the institutional body and having worked at the institution for more than six months. Exclusion was based on being on vacation or sick leave during the data collection period. Data was collected through semi-structured interviews. To ensure comfort and anonymity, the interviews were conducted in the units’ consulting rooms. They were recorded and transcribed for later analysis. The letters NUR were used to identify the participants’ statements, followed by the number of the interviews. The nurses were interviewed during their shifts, on the day and at the time they had chosen. In the FHS, care for the population begins at 8 am, so these varied according to their availability.

### Data collection and organization

The data was collected between December 2022 and January 2023, through individual interviews with a semi-structured questionnaire, conducted by the main researcher. He was an undergraduate nursing student with prior training in conducting interviews, which took place in a room provided in the unit where the nurse worked. They were conducted during working hours and audio-recorded, lasting around 60 minutes.

The guiding question was “What strategies do you use to deal with underreporting of pesticide use in rural areas?”. In addition, all the participants had the opportunity to listen to their interview, to make comments or correct information, but no nonconformities were pointed out.

A pilot test was carried out in a primary care unit and a secondary care unit, involving five nurses who treated rural workers with exogenous poisoning from pesticide use resulting from accidents at work. As there was no need to adjust the semantics and structure of the guiding questions during the interview, they were incorporated into the research corpus.

Data collection ended when the researcher had achieved the objective proposed in the study. The quality of the acts and interactions is sought and reflected in an ideal qualitative sample, with the researcher’s conviction of having discovered the internal logic of their object of study prevailing^(15)^.

### Data analysis

The content analysis technique, described by Bardin, is a set of research methods aimed at interpreting the evident content of communications by means of a systematic, objective, and quantitative description^(16)^. This technique was used to analyze the data, which takes place in three stages. The first is the data organization phase, in which documents are selected for analysis, assumptions and objectives are formulated, and categories are created. The second stage is the exploration of the material, in which the text is systematically analyzed according to the categories created previously. The third stage is the treatment and inference of the results.

## RESULTS

**Figure 1:**
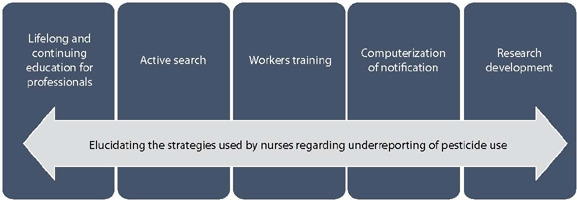
Diagram of the categories that emerged from the study

Data analysis revealed the characterization of the nurses participating in the study and the categories presented, as shown in Figure 1.

The participants were all female, aged between 25 and 51, and had worked in primary and secondary health care for between two and 15 years. The strategies used by nurses regarding underreporting of pesticide use in rural areas are presented below.

### Carrying out lifelong and continuing education for professionals who (should) report accidents

Continuing education was the strategy most mentioned by the nurses participating in the study. Training and capacity-building for the teams are essential if notifications are to be made correctly, feeding the information system.

[...] *I see the relevance of continuing education with other health team members.* (NUR11)

*I think it’s important to train the teams on the SINAN forms, showing them how to fill them in. We need to train the professionals who carry out screening or reception to identify when to make these notifications.* (NUR07)

*There is a need for lifelong education for the units and training for staff on how to fill in the notifications.* (NUR08)

*The strategy I would use is to train everyone on the team. Trying to find articles on the subject, for example, to train the team so that when a case of exogenous poisoning due to pesticide use arrives, everyone knows what to do*. (NUR09)

*One possibility would be to attend lectures on the subject.* (NUR18)

*Continuing education for the entire nursing team and other health professionals on the importance of reporting so that public health policies are effective in dealing with the health problems of the population exposed to pesticides.* (NUR20)

### Active search of workers

Actively seeking out these workers is also a key factor in promoting and preventing accidents at work in this population, which lives furthest away from health services. Active search would make it possible to better collect data, encourage the use of PPE, and the seeking of health services in the event of an accident at work.

*The active search of cases can ensure adequate data collection.* (NUR05)

*Actively seeking out these people. Talking to and advising rural workers to seek health services in cases of poisoning, so that they can be notified. In addition, the correct use of PPE. These are strategies that provide support for prevention and health promotion.* (NUR17)

*It is necessary to intensify the work of surveillance agencies to show how important it is to report the disease correctly. To do this, they would use active search and home visits in rural areas.* (NUR19)

### Training of workers who deal directly with this type of substance

It is of the utmost importance to train workers who deal directly with this type of substance. Farmers often fail to use PPE, leading to long-term exogenous poisoning. This educational process takes place through talks, not only in the places where the work is carried out, but also in communities and basic health units, where the professional provides pertinent information on how to protect oneself, how to handle this type of substance and even how to apply the product. Many people do not know that the pesticide should be applied in the direction of the wind, so that it is not aspirated during the procedure. They also mentioned that the workers become multipliers of knowledge for other farmers they work with.

*Health education for workers is very important. Get the staff together and train them in worker safety, and try to make them aware that they don’t have to be afraid to report an accident at work to a health professional. There will be no retaliation. Also, that there is a health professional who wants to create a safe working environment for the worker and the company.* (NUR02)

*One strategy to be adopted is to advise on the importance of reporting.* (NUR03)

*It would be interesting to make a folder with all the information workers need on how to apply and handle these products to avoid accidents caused by pesticide poisoning. It would also be useful to inform them of the importance of reporting accidents at work.* (NUR10)

*Training for the workers themselves, as they disseminate this information so that other workers have an idea of what they are working with and what kind of symptoms they may experience in the event of exogenous poisoning from long-term pesticide use.* (NUR14)

*Home visits are a very important strategy in this situation. I would use health education with people who work at street markets as a strategy, because most of the time these people are farmers who deal directly with pesticides in their crops. By educating these people and making them aware of the risks they run, I believe they would try to protect themselves first and foremost.* (NUR15)

### Computerization of notification through online forms

Notifications of accidents at work are mandatory and must be made in order to feed the information system and epidemiological surveillance, and their completion is of the utmost importance for health professionals. The nurses said that computerizing these notifications would reduce the time it takes to fill them out, even more so if the forms were linked to patients’ medical records, as patients’basic information would already be filled in automatically, reducing the time it takes to fill them out. Hence, the notification structure is no longer on paper but online, integrating all the patients’ information into a single system, making it a more objective tool that can be accessed anywhere.

*The computerization of health systems currently makes it possible to fill out patient records online.* (NUR04)

*I believe that many of the services have access to SINAN, in which the specific notification allows us to guide data collection more easily.* (NUR06)

*Some notifications have entered the system. This has made it much easier, because we can mark the SINAN fields on the notification form. For example: accident at work, the form is in the system. It’s easier.* (NUR12)

*The way notification is carried out is very simple, straightforward, and effective. We have specific epidemiological surveillance forms for each thing. You go into the system and just use that form, fill it in, and send it to epidemiological surveillance.* (NUR13)

*Raising awareness of the importance of notifications for nurses.* (NUR 16)

*Filling in the form on the SINAN website. Linking notifications to the patient’s medical record, so that basic information is filled in automatically when you click on a notification form, making it easier and quicker to fill in the form.* (NUR20)

### Carrying out research on the subject

The professionals interviewed reported the importance of further research on the subject. They suggest that knowledge about occupational accidents related to exogenous intoxication as a result of pesticide use in the rural environment should be encouraged.

*Policies and education need to go hand in hand. Knowledge needs to be reinforced within universities, so that professionals who are going to enter the job market have this kind of approach and are able to get those who work in these regions to seek training in universities, or from political management, to be able to deal with this kind of notification.* (NUR01)

*I also think it’s important to carry out academic research that shows how damaging underreporting is for rural workers themselves. This is because if there is no information about the problems, nothing is done, no policy is developed for them.* (NUR07)

## DISCUSSION

The nurses mentioned the following as strategies for reporting accidents at work: lifelong and continuing education for professionals who report accidents and active search of workers. In addition, the training of workers who deal directly with this type of substance, the computerization of notification by filling in online forms, and researching the subject are indispensable, as observed in this study.

Regarding the continuing education of health professionals who submit the notifications, many who work in primary and emergency care units are unaware of what they should notify, when to notify, and which forms should be used. In order to minimize this problem, improvements should be made in the reporting of exogenous poisoning due to pesticide use, considering that there are still gaps in the care provided to rural workers, making them vulnerable to this problem^(17)^.

Professionals should be trained. Educational strategies aimed at health professionals who work directly with occupational accident notifications should be implemented as a form of continuing education, providing better knowledge to carry out all types of notifications^(17)^.

Concerning active search, nurses are one of the professionals responsible for comprehensive care in primary healthcare interventions, so that user involvement is not only focused on the disease, but also on working and living conditions^(18)^. Nurses play an important role in diagnosing exogenous poisoning caused by the use of pesticides in rural environments. The potential for poisoning is identified during the nursing consultation and/or reception, according to the type of exposure and the symptoms presented. In view of the above, it is important for nurses to be able to collect information relevant to the patient’s case, and for each case to promote appropriate intervention actions, using data collection at the place where these workers carry out their work as a fundamental tool for quality care, notifying cases of exogenous poisoning on SINAN^(19)^.

Proper notification in the workplace is essential to ensure that workers receive the care and support they need to recover safely and healthily. Health professionals are responsible for identifying, evaluating, and treating occupational accidents and diseases. In addition, they must report these incidents to the relevant authorities to ensure accurate occupational accident statistics and preventive measures^(20)^.

Training rural workers is necessary, as many are unaware that exogenous poisoning due to the use of pesticides is a common problem. As a result, misinformation about the risks and negative effects of chemical products and other toxic substances used in rural areas contributes to their indiscriminate use. A study found that many problems related to exogenous intoxication of rural workers occur due to a lack of knowledge about the necessary precautions when handling these substances. In particular, incorrect use of PPE and handling of substances, both in their storage, manipulation and application, lead to unnecessary exposure to chemical products^(21)^.

Educational practice enables these workers to reflect on their work. Many of these workers have a relatively low level of schooling, which generates a number of problems that will have a direct impact on their health and safety. Health professionals, together with rural workers, can contribute to preventing the impacts and harm caused by the indiscriminate use of pesticides in the rural environment^(22)^.

It is important to carry out informal health education in a language that workers understand. Awareness of how to handle pesticides and how to apply them correctly is essential, as is the use of PPE to avoid accidents at work. Raising awareness among the population is important for reducing exogenous poisoning, in terms of proper handling, correct disposal, as well as seeking care in health services, a priori, in primary health care, in the event of any manifestation of symptoms that characterize poisoning. This work can be done with the support of health teams, carrying out this education in schools, fairs, and health units^(4)^.

Incorrect handling, storage, and application often cause these workers to suffer this type of accident at work. Many of them cannot read, so the instructions on the packaging go unnoticed. The population of rural workers, especially those who deal directly with pesticides, have a low perception of the risks arising from exposure to these substances^(23)^.

One way of facilitating the notification of accidents at work is to computerize notifications, since an information system integrated with patients’ medical records would help speed up the work process. The computerization of notifications has proven to be an effective way of optimizing communication processes in various sectors, which increases the efficiency of information exchange. There is a need to implement an information system that can guarantee the reliability of data collection, storage, and systematization of information in patients’ medical records, so that the reality of the population studied can be brought to life and the necessary information can be accessed anywhere^(4)^.

Therefore, research into the repercussions of pesticides on environmental matrices and on the health of workers, the population and farming communities is essential and is one of the mechanisms for public health surveillance^(12)^. It is necessary to develop new research on the subject, in order to develop a population base for understanding the effects of pesticide poisoning on populations in different regions of the country, knowing the intrinsic characteristics of these poisonings, especially in the regions with the highest growth in these diseases^(24)^.

### Study limitations

The limitation of this study was that it only focused on nurses’ perspectives. This study is expected to become a reference for research that addresses the issue of underreporting of exogenous poisoning secondary to the use of pesticides in the rural environment, from the perspective of these professionals, making it possible to approach the issue from other points of view.

### Contributions to the field of Nursing, Health or Public Policy

The study provides relevant information about the underreporting of exogenous intoxication among rural workers, which is often detrimental to health information systems. In this sense, the strategies listed by the professionals allow for a reflection-action on what is currently done and how this process can be improved, with the aim of reducing underreporting and, consequently, directing public policies to raise awareness among the rural population about the risks of incorrect or mistaken handling of substances such as pesticides. In addition, nurses, as front-line agents working in primary and secondary care units, are faced with the immediate situation, so it is essential that they know how to recognize the signs and symptoms, carry out a thorough assessment based on the patient’s individuality in order to make a correct diagnosis and, subsequently, notify the case appropriately.

## FINAL CONSIDERATIONS

The strategies used by nurses regarding underreporting of pesticide use in rural areas are an important tool for public health. Nurses, as professionals who work directly in the health care of rural workers, play an important role in notifying occupational accidents caused by the use of pesticides. In order to improve this process, they need to implement effective strategies such as keeping up to date, participating in continuing and lifelong education programs on the subject, training rural workers who use these substances in their work, so that they understand the importance of reporting accidents, actively searching for them in their workplaces, reporting on computerized forms, in a system integrated with the patient’s information, facilitating the process and contributing to new research, ensuring up-to-date knowledge.

Reporting cases correctly can strengthen health policies and improve worker safety in rural areas. Specific educational actions and training for these professionals are necessary, in order to increase their awareness of the risks related to pesticide use and their responsibility for reporting and preventing these accidents. Furthermore, collaboration between nurses and rural workers is an effective strategy for preventing and controlling exogenous poisoning. Exchanging knowledge contributes to improving the lives of workers exposed to pesticide use, and has the potential to reduce the social and economic costs associated with these accidents.
