# Supplementary material for: Strategies used by nurses regarding underreporting of rural work accidents due to pesticide use
Source: Rev Bras Enferm. 2024 Jun 14;77(2):e20230384. doi: 10.1590/0034-7167-2023-0384 (PMC11178317; doi:10.1590/0034-7167-2023-0384)
Supplement: Translation

# Translation: Estratégias utilizadas por enfermeiros sobre subnotificações de acidentes de trabalho rural por uso de agrotóxicos

**DOI:** 10.1590/0034-7167-2023-0384pt

**Authors:** Dionatan Carmini de Brito, Daiani Modernel Xavier, Alberto de Oliveira Redü, Luciano Garcia Lourenção, Franciele Gomes Soares, Giovana Calcagno Gomes, Laura Fontoura Perin

**Affiliations:** I Universidade Federal do Rio Grande. Rio Grande, Rio Grande do Sul, Brasil

**Keywords:** Sub-Registro, Saúde Ocupacional, Acidentes de Trabalho, Enfermagem, Zona Rural

## Abstract

**Objetivo:**

Conhecer as estratégias utilizadas acerca das subnotificações por uso de agrotóxicos no meio rural.

**Método:**

Estudo qualitativo realizado em oito unidades básicas de regiões rurais e duas unidades de pronto atendimento de um município do sul do Brasil. A coleta de dados deu-se em 2023, por entrevistas. Participaram 20 profissionais enfermeiros. Os dados foram submetidos à análise de conteúdo.

**Resultados:**

Apontaram como estratégias a realização da educação permanente e continuada dos profissionais que realizam a notificação, a busca ativa e a capacitação dos trabalhadores que lidam diretamente com esse tipo de substância, a informatização da notificação por meio do preenchimento das fichas online e a realização de pesquisas acerca da temática.

**Considerações Finais:**

Os enfermeiros desempenham um papel importante na notificação de acidentes de trabalho pelo uso de agrotóxicos, melhorando a segurança ocupacional no meio rural.

## INTRODUÇÃO

O panorama do espaço agrário no Brasil vem sofrendo grandes modificações que estão diretamente ligadas ao projeto de modernização da agricultura, especialmente, com a reestruturação da produção no campo. Desse modo, ocorreu a criação de políticas agrícolas voltadas para a modernização de algumas regiões do Brasil, para que a produção das monoculturas pudesse atender às agroindústrias e às demandas do mercado externo, ocorrendo o uso em massa de agrotóxicos^(1)^.

Os agrotóxicos, também conhecidos como defensivos agrícolas, pesticidas ou praguicidas, apresentam aspectos positivos relacionados ao processo de trabalho agrícola que permitem proteger lavouras e plantações contra pragas, além de viabilizar essas lavouras em larga escala. No entanto, se mal manuseados, causam consequências negativas para a saúde da população exposta^(2)^. A Organização Internacional do Trabalho (OIT) aponta que mais de 70 mil intoxicações agudas e crônicas por ano ocorrem em decorrência do uso de agrotóxicos, muitas vezes, evoluindo a óbitos nos países desenvolvidos, onde mais de sete milhões de casos são notificados e, em países em desenvolvimento, como o Brasil, o consumo de agrotóxico no setor agrícola apresenta aumento significativo^(3)^.

Segundo a OIT, cerca de 70.000 trabalhadores, em países em desenvolvimento, morrem de intoxicação aguda e/ou crônica por intoxicação exógena, ocasionada pelo uso de agrotóxicos, outros sete milhões de pessoas são acometidas por doenças não fatais relacionadas a essa prática. A intoxicação por agrotóxicos pode ocorrer por contato com o trato digestivo, trato respiratório, pele e/ou olhos e pode ser aguda, subaguda ou crônica. Entre os grupos mais afetados, estão os agricultores e pecuaristas expostos a esse tipo de substância durante o preparo dos produtos e pelo uso direto, sem o uso dos Equipamentos de Proteção Individual (EPIs), e durante o uso das embalagens após o manuseio^(4)^.

A forma mais comum de contaminação é a exposição direta através do meio ambiente, atingindo grande parte da população. Os principais efeitos à saúde podem ser agudos, sendo aqueles que têm o aparecimento dos seus sintomas de forma rápida, podendo surgir desidratação, irritação na pele, alergias, ardência no nariz e na boca, coriza, tosse, dor no peito, dificuldade para respirar, dor de garganta, dor de estômago, náuseas, vômitos e diarreia. Outros sintomas considerados inespecíficos podem surgir, como cefaleia, fraqueza, transpiração anormal, tremores e irritabilidade^(5)^.

Alguns grupos de agrotóxicos são causadores de câncer em indivíduos que entram em contato frequente, sendo necessárias precauções para o seu manuseio^(6)^. A intoxicação exógena por uso de agrotóxicos é considerada um problema de saúde pública pelo Ministério da Saúde e pela Organização Mundial da Saúde (OMS), sendo agravado pelas subnotificações que ocasionam um *déficit* informacional no sistema brasileiro. Nesse sentido, para cada registro de intoxicação por agrotóxicos realizado, há outros 50 não notificados^(7)^.

Com a aprovação do Ministério da Agricultura para a liberação de novos agrotóxicos, o aumento de novos registros de intoxicação de trabalhadores apontou uma continuidade acelerada dessa tendência. Segundo o Sistema de Informação de Agravos de Notificação (SINAN), entre 2007 e 2017, verificaram-se 41,6 mil casos de intoxicação decorrentes do manuseio incorreto ou inadequado de agrotóxicos, principalmente na zona rural^(8)^.

Diante disso, destaca-se a importância das notificações desses acidentes de trabalho. No Brasil, foram criadas Normas Regulamentadoras (NR), a *priori*, a NR 6, para garantir a prevenção contra acidentes dessa natureza, passando-se a fiscalizar mais a utilização dos EPIs pelos trabalhadores que manuseiam esses produtos^(9)^.

Em decorrência, ao ocorrer um acidente de trabalho, torna-se importante atentar para o preenchimento da ficha de notificação compulsória, muitas vezes, de responsabilidade do enfermeiro. Essas informações de saúde do trabalhador destinam-se a ser utilizadas na comunidade, para monitorar os casos e tomar medidas para promover, proteger e restabelecer a saúde no local de trabalho^(10)^.

No entanto, as subnotificações ainda são uma realidade no ambiente de trabalho, e cabe às instituições, por meio de seus comitês, estimular um processo de reflexão sobre a sua importância. Sublinha-se que a rede pública de saúde relata mais a ocorrência de acidentes do que as redes privadas, o que serve tanto para prevenir quanto para controlar esses eventos, protegendo a saúde e a segurança dos trabalhadores^(10)^.

A partir disso, em decorrência dos sintomas imprecisos relatados pelos agricultores e a baixa procura dos serviços de saúde, ocorre a subnotificação das intoxicações exógenas por uso de agrotóxicos. Nos municípios pequenos, a desinformação torna-se um fator agravante e, nesse sentido, buscar estratégias por meio da visão dos profissionais de saúde, em especial os enfermeiros, que contribuam para informações com o intuito de promover saúde e prevenir agravos da população exposta transforma-se em um objetivo comum enfrentado pelos serviços de saúde^(11)^.

Os profissionais de saúde devem ser treinados para reconhecer as situações de risco, e as empresas devem tomar medidas para ajudar a atingir esses objetivos, buscando reduzir a subnotificação de acidentes de trabalho. Embora o Brasil tenha um dos melhores Sistemas de Informação em Saúde (SIS), sendo capaz de registrar lesões e doenças ocorridas no território nacional, em decorrência de problemas causados or agrotóxicos, dependendo do grau de exposição à saúde, muitas vezes, ocorrem subnotificações. Há dificuldades dos profissionais determinarem a relação causal das intoxicações por agrotóxicos, cujas observações são importantes para fundamentar ações interdisciplinares^(12)^. Nesse contexto, torna-se importante criar estratégias para prevenir as subnotificações dos acidentes de trabalho causados por intoxicações por agrotóxicos. Assim, o presente estudo teve a seguinte questão norteadora: Quais as estratégias utilizadas por enfermeiros sobre subnotificações de acidentes de trabalho rural por uso de agrotóxicos?

## OBJETIVO

Conhecer as estratégias utilizadas por enfermeiros sobre sub-notificações de acidentes de trabalho rural por uso de agrotóxicos.

## MÉTODOS

### Aspectos Éticos

Seguiu-se a Resolução da Comissão Nacional de Ética em Pesquisa/Ministério da Saúde (CONEP/MS) 466/2012 sobre pesquisas com humanos^(13)^. A natureza, objetivos e métodos da pesquisa foram explicados aos participantes. Os que concordaram em participar assinaram o Termo de Consentimento Livre e Esclarecido (TCLE), em duas vias, ficando uma cópia com o participante e a outra com o pesquisador.

### Tipo de Estudo

Estudo do tipo descritivo, exploratório de natureza qualitativa. Com o intuito de qualificar a produção científica acerca da temática, foram adotadas as diretrizes do *Consolidated Criteria for Reporting Qualitative Research* (COREQ)^(14)^.

### Cenário do Estudo

O cenário deste estudo consistiu-se em oito Unidades de Estratégia Saúde da Família (ESF), localizadas em áreas rurais, e duas Unidades de Pronto Atendimento. As unidades rurais destinam-se ao atendimento primário dos casos, porém tornam-se indisponíveis nos finais de semana e feriados. Por outro lado, as unidades de pronto atendimento são vistas como atendimento primário, quando há indisponibilidade das unidades rurais, e secundário para fornecer apoio necessário para a continuidade do atendimento, quando esse se inicia na ESF. As unidades mencionadas acima estão localizadas em um município da região sul do sul do Rio Grande do Sul, Brasil, e fornecem atendimentos integrais à população de acordo com as diretrizes do Sistema Único de Saúde (SUS).

### Fonte de dados

Participaram vinte enfermeiros que prestavam assistência. Os requisitos para ser incluído foram: enfermeiro devidamente certificado pelo órgão institucional e ter trabalhado na instituição por mais de seis meses. A exclusão foi baseada em estar em férias ou licença saúde no período da coleta de dados. Os dados foram coletados por meio de entrevistas semiestruturadas. Para garantir conforto e anonimato, as entrevistas foram realizadas em consultórios das unidades. Foram gravadas e transcritas para posterior análise. As letras ENF foram usadas para identificar as falas dos participantes, que foram seguidas pelo número de realização das entrevistas. Os enfermeiros foram entrevistados durante seus turnos de trabalho, no dia e horário que eles haviam escolhido. Na ESF, o atendimento à população começa às 8h, então estes variaram de acordo com a disponibilidade de cada um.

### Coleta e organização dos dados

A coleta ocorreu entre dezembro de 2022 e janeiro de 2023, por meio de entrevistas individuais com questionário semiestruturado, realizado pelo pesquisador principal, graduando do curso de enfermagem, com treinamento prévio para condução de entrevista, conduzida em sala cedida pela própria unidade onde o enfermeiro trabalhava. Foram realizadas durante o período de trabalho e gravadas em áudio, com duração de cerca de 60 minutos.

Como questão norteadora adotou-se “Quais as estratégias utilizadas por você acerca das subnotificações por uso de agrotóxicos no meio rural?” Além disso, todos os participantes tiveram a oportunidade de ouvir sua entrevista, para indicar comentários ou corrigir informações, contudo, nenhuma inconformidade foi apontada.

Realizou-se teste piloto em uma unidade de atenção primária e uma de atenção secundária, abrangendo cinco enfermeiros que atenderam trabalhadores rurais com intoxicação exógena por uso de agrotóxicos, decorrentes de acidentes de trabalho. Por não apresentar necessidade de adequação semântica e estrutural das questões norteadoras durante a entrevista, estas foram incorporadas ao corpo da pesquisa.

Ocorreu o encerramento da coleta de dados, quando o pesquisador alcançou o objetivo proposto no estudo. A qualidade dos atos e das interações é buscada e refletida em uma amostra qualitativa ideal, prevalecendo a convicção do pesquisador de ter descoberto a lógica interna do seu objeto de estudo^(15)^.

### Análise dos dados

A técnica de Análise de Conteúdo, descrita por Bardin, é um conjunto de métodos de pesquisa que visam à interpretação do conteúdo evidente das comunicações por meio de uma descrição sistemática, objetiva e quantitativa^(16)^. Essa técnica foi usada para analisar os dados. A análise é feita em três etapas. A primeira é a fase de organização dos dados, na qual os documentos são selecionados para a análise, os pressupostos e os objetivos são formulados e as categorias são criadas. A segunda etapa é a exploração do material, na qual o texto é analisado sistematicamente em função das categorias criadas anteriormente. A terceira etapa é o tratamento e a inferência dos resultados.

## RESULTADOS

A análise dos dados elucidou a caracterização dos enfermeiros participantes do estudo e as categorias apresentadas, conforme a Figura 1.

**Figura 1:** Diagrama das categorias que emergiram do estudo

As participantes foram todas do sexo feminino, com idades entre 25 e 51 anos e período de atuação de dois a 15 anos na Atenção Primária à Saúde (APS) e Secundária. A seguir, foram apresentadas as estratégias utilizadas por enfermeiros acerca das subnotificações por uso de agrotóxicos no meio rural.

### Realização da educação permanente e continuada dos profissionais que realizam (devem realizar) a notificação do acidente

A educação permanente foi a estratégia mais elencada pelas enfermeiras participantes do estudo. O treinamento e a capacitação das equipes tornam-se fundamentais para que as notificações sejam realizadas de forma correta, alimentando o sistema de informação.

[...] *Percebo relevante a educação permanente com os demais colaboradores da equipe de saúde.* (ENF11)

*Acho importante fazer uma capacitação com as equipes sobre as fichas do SINAN, mostrando como preenchê-las. É preciso realizar uma capacitação com os profissionais que realizam a triagem ou acolhimento para identificar quando fazer essas notificações.* (ENF07)

*É preciso educação permanente para as unidades e treinamento para o pessoal como preencher as notificações.* (ENF08)

*A estratégia que eu utilizaria é o treinamento de todos da equipe. Tentar buscar artigos acerca da temática, por exemplo, para treinar a equipe para que, quando chegar um caso de intoxicação exógena por uso de agrotóxico, todos saibam fazer*. (ENF09)

*Uma possibilidade seria a participação de palestras sobre o tema.* (ENF18)

*Educação continuada de toda equipe de enfermagem e demais profissionais da saúde sobre a importância das notificações, para que as políticas públicas de saúde sejam eficazes diante do agravo de saúde da população exposta ao agrotóxico.* (ENF20)

### Busca ativa dos trabalhadores

A busca ativa desses trabalhadores também é um diferencial, visando à promoção e prevenção dos acidentes de trabalho, nessa população que reside mais distante dos serviços de saúde. A busca ativa possibilitaria uma melhor coleta dos dados, o incentivo ao uso dos EPIs e a procura dos serviços de saúde em caso de acidente de trabalho.

*A busca ativa dos casos pode garantir uma coleta de dados adequada.* (ENF05)

*Busca ativa dessas pessoas. Conversar e orientar os trabalhadores rurais a procurarem os serviços de saúde em casos de intoxicação, para a devida notificação. Além disso, o uso correto de EPI. São estratégias que fornecem subsídios para prevenção e promoção da saúde.* (ENF17)

*Torna-se necessário o trabalho intensificado por parte das vigilâncias, para mostrar o quanto se faz importante uma notificação correta. Para isso, usaria a busca ativa e as visitas domiciliares nas áreas rurais.* (ENF19)

### Capacitação dos trabalhadores que lidam diretamente com esse tipo de substância

É de suma importância realizar a capacitação dos trabalhadores que lidam diretamente com esse tipo de substância. Muitas vezes, é possível observar que os agricultores não utilizam os EPIs, levando-os à intoxicação exógena a longo prazo. Esse processo educativo se dá por meio de conversas, não só nos locais onde é exercido o trabalho, mas também nas comunidades e nas unidades básicas de saúde, no qual o profissional leva informações pertinentes de como se proteger, como manusear esse tipo de substância e, até mesmo, a maneira de aplicar o produto. Muitas pessoas não sabem que o agrotóxico deve ser aplicado na direção do vento, para que ele não seja aspirado durante o procedimento. Referiram, também, que os trabalhadores se tornam multiplicadores de conhecimentos para outros agricultores com quem convivem.

*É muito importante uma educação dos trabalhadores para a saúde. Reunir o pessoal e fazer uma capacitação sobre a segurança do trabalhador, além de tentar conscientizar de que não precisa ter medo de comunicar o acidente de trabalho ao profissional de saúde. Não vai haver represália. Além disso, que existe um profissional de saúde querendo criar um ambiente seguro de trabalho para o trabalhador e para empresa.* (ENF02)

*Uma estratégia a ser adotada é a orientação da importância da notificação.* (ENF03)

*Seria interessante fazer um folder com todas as informações para que o trabalhador tivesse de como aplicar e manusear esses produtos, a fim de evitar acidentes, em decorrência da intoxicação por uso do agrotóxico. Também, informar sobre a importância de realizar as notificações em caso de acidentes de trabalho.* (ENF10)

*Capacitação para o próprio trabalhador, pois ele dissemina essas informações, para que outros trabalhadores tenham uma noção do que eles estão trabalhando e que tipo de sintomas eles podem apresentar em caso de intoxicação exógena por uso de agrotóxicos a longo prazo.* (ENF14)

*A visita domiciliar é uma estratégia muito importante nessa situação. Utilizaria como estratégia a educação em saúde com as pessoas que trabalham nas feiras, porque quem trabalha nesses locais, na maioria das vezes, são agricultores que lidam direto com o agrotóxico nas suas plantações. Ao educar essas pessoas e a ter esse conhecimento sobre quais os riscos que elas correm, acredito que procurariam se proteger em primeiro lugar.* (ENF15)

### Informatização da notificação por meio do preenchimento das fichas online

As notificações de acidentes de trabalho são obrigatórias e devem ser feitas para alimentar o sistema de informação e a vigilância epidemiológica, sendo o seu preenchimento pelos profissionais da saúde de suma importância. As enfermeiras referiram que a informatização dessas notificações diminuiria o tempo de preenchimento, ainda mais se a ficha estivesse interligada ao prontuário do paciente, pois as informações básicas deste já estariam preenchidas de forma automática, diminuindo o tempo do preenchimento. Desse modo, a estrutura das notificações sai do papel e passa a ser online, integrando todas as informações do paciente em um único sistema, tornando-se uma ferramenta mais objetiva, podendo ser acessada de qualquer lugar.

*Hoje com a informatização dos sistemas de saúde, é possível o preenchimento online já com as informações contidas nas fichas dos pacientes.* (ENF04)

*Acredito que muitos dos serviços dispõem de acesso ao SINAN, no qual a notificação específica nos permite nortear a coleta de dados de forma mais facilitada.* (ENF06)

*Tem algumas notificações que entraram para o sistema. Foi uma coisa que facilitou muito, porque vamos marcando os campos do SINAN na ficha de notificação. Por exemplo: acidente de trabalho, a ficha está no sistema. Fica uma coisa mais fácil.* (ENF12)

*A forma de executar a notificação é bem simples, direta e efetiva. A gente tem formulários específicos da vigilância epidemiológica para cada coisa. Vai no sistema e só vai usar aquele formulário, preencher e encaminhar para a vigilância epidemiológica.* (ENF13)

*Trazer a importância das notificações para o enfermeiro.* (ENF16)

*O preenchimento da ficha no site do SINAN. A interligação das notificações junto ao prontuário do paciente, fazendo com que as informações básicas sejam preenchidas, de forma automática, ao clicar em uma ficha de notificação, facilitando o tempo e agilizando o preenchimento do formulário.* (ENF20)

### Realização de pesquisas acerca da temática

Os profissionais entrevistados relataram a importância de novas pesquisas sobre a temática. Sugerem que seja estimulado o conhecimento sobre os acidentes de trabalho relacionados à intoxicação exógena em decorrência do uso de agrotóxico no ambiente rural.

*As políticas e a educação precisam caminhar juntos. É necessário reforçar o conhecimento dentro das universidades, para que os profissionais que irão ingressar no mercado de trabalho tenham esse tipo de olhar e consigam fazer com que os próprios profissionais que trabalham nessas regiões busquem, junto às universidades ou à gestão política, o treinamento para poder lidar com esse tipo de notificação.* (ENF01)

*Também acho importante a realização de pesquisas acadêmicas que mostrem o quanto é prejudicial essas subnotificações para os próprios trabalhadores rurais. Isso ocorre porque se não há informação dos problemas, nada é feito, nenhuma política é desenvolvida para os mesmos.* (ENF07)

## DISCUSSÃO

Os enfermeiros referiram, como estratégias para realizar as notificações dos acidentes de trabalho, a realização da educação permanente e continuada dos profissionais que realizam (devem realizar) a notificação do acidente e a busca ativa dos trabalhadores. Além disso, a capacitação dos trabalhadores que lidam diretamente com esse tipo de substância, a informatização da notificação por meio do preenchimento das fichas *online* e a realização de pesquisas acerca da temática são indispensáveis, neste estudo.

No que se refere à educação continuada dos profissionais de saúde que realizam as notificações, muitos que atuam nas unidades básicas e unidades de pronto-atendimento desconhecem o fato de o que se deve notificar, em qual momento notificar e quais os formulários devem ser utilizados. Com vistas a minimizar esse problema, deve-se buscar aprimoramento acerca das notificações de intoxicações exógenas por uso de agrotóxicos, considerando-se que ainda há lacunas nas ações de cuidado ao trabalhador rural, tornando-os vulneráveis a essa problemática^(17)^.

Os profissionais devem realizar capacitações e treinamentos. Estratégias educacionais voltadas aos profissionais da saúde que atuam diretamente com as notificações de acidente de trabalho devem ser implementadas como forma de educação continuada, proporcionando um melhor conhecimento para realizar todos os tipos de notificações^(17)^.

Em relação à busca ativa, o enfermeiro é um dos profissionais responsáveis pela assistência integral nas intervenções da APS, de modo que o envolvimento do usuário não é apenas focado na doença, mas também nas condições de trabalho e vida^(18)^. O enfermeiro desempenha um trabalho importante no diagnóstico das intoxicações exógenas causadas pelo uso de agrotóxicos nos ambientes rurais. A partir da consulta de enfermagem e/ou no acolhimento, é que se identifica a potencialidade da intoxicação, de acordo com o tipo de exposição e sintomas apresentados. Diante do exposto, é importante que o enfermeiro consiga coletar informações relevantes no caso do paciente e, para cada caso, promover ações de intervenção adequadas, utilizando, como instrumento fundamental para assistência de qualidade, a coleta de dados no local onde esses trabalhadores executam o seu trabalho, realizando a notificação dos casos de intoxicação exógena no SINAN^(19)^.

A notificação adequada no local de trabalho torna-se essencial para garantir que os trabalhadores recebam os cuidados e o apoio de que precisam, para se recuperar com segurança e saúde. Os profissionais de saúde são responsáveis por identificar, avaliar e tratar acidentes e doenças ocupacionais. Além disso, devem relatar esses incidentes às autoridades competentes, para garantir a precisão das estatísticas de acidentes de trabalho e medidas preventivas^(20)^.

A capacitação dos trabalhadores rurais se faz necessária, visto que muitos desconhecem que as intoxicações exógenas em decorrência do uso de agrotóxicos são um problema comum. A partir disso, a desinformação sobre os riscos e efeitos negativos dos produtos químicos e outras substâncias tóxicas utilizadas no meio rural contribui para seu uso indiscriminado. Estudo constatou que muitos problemas relacionados à intoxicação exógena desses trabalhadores rurais ocorrem em decorrência da falta de conhecimento sobre os cuidados necessários com o manuseio dessas substâncias. Destacam-se o uso incorreto dos EPIs e o manuseio incorreto das substâncias, tanto no seu armazenamento como na manipulação e aplicação, que acarretam a exposição desnecessária aos produtos químicos^(21)^.

A prática educativa possibilita a reflexão desses trabalhadores acerca do seu fazer profissional. Muitos desses trabalhadores apresentam um nível de escolaridade relativamente baixo, o que gera diversos prejuízos, o que irá influenciar diretamente na sua segurança e saúde. Os profissionais da saúde, juntamente com os trabalhadores rurais, podem contribuir para a prevenção dos impactos e agravos do uso indiscriminado dos agrotóxicos no ambiente rural^(22)^.

É importante realizar uma educação em e para a saúde de maneira informal, em uma linguagem que os trabalhadores compreendam. A conscientização do manuseio e a forma correta de aplicação dos agrotóxicos tornam-se indispensáveis, bem como o uso dos EPIs, a fim de se evitarem acidentes de trabalho. A conscientização da população é importante para a redução das intoxicações exógenas, no que concerne à forma adequada de manejo, ao descarte correto, bem como à busca pelo atendimento nos serviços de saúde, a *priori*, na APS, diante de qualquer manifestação de sintomas que caracterize uma intoxicação. Esse trabalho pode ser concretizado com o apoio das equipes de saúde, realizando essa educação em escolas, feiras e unidades de saúde^(4)^.

O manuseio, armazenamento e a forma de aplicação incorreta, muitas vezes, fazem com que esses trabalhadores sofram esse tipo de acidente de trabalho. Muitos deles não sabem ler, sendo assim, as instruções contidas nas embalagens passam despercebidas. A população de trabalhadores rurais, em especial aqueles trabalhadores que lidam diretamente com agrotóxicos, possui baixa percepção dos riscos em decorrência da exposição a essas substâncias^(23)^.

Um elemento facilitador da realização da notificação do acidente de trabalho referido é a informatização das notificações, uma vez que o sistema de informação integrado com o prontuário do paciente contribuiria para a agilização do processo de trabalho. A informatização das notificações tem se mostrado uma forma eficaz de otimizar os processos de comunicação em diversos setores, o que aumenta a eficiência na troca de informações. Há necessidade da implementação de um sistema de informação que possa garantir uma confiabilidade na coleta de dados, no armazenamento e na sistematização das informações no prontuário dos pacientes, podendo trazer a realidade da população estudada e a possibilidade de acesso às informações necessárias em qualquer lugar^(4)^.

Portanto, a realização de pesquisas acerca das repercussões dos agrotóxicos nas matrizes ambientais e na saúde dos trabalhadores, da população e das comunidades agrícolas torna-se fundamental e constitui um dos mecanismos de vigilância em saúde pública^(12)^. Faz-se necessário o desenvolvimento de novas pesquisas relacionadas à temática, a fim de desenvolver uma base populacional para a compreensão dos efeitos das intoxicações por agrotóxicos nas populações de diferentes regiões do país, conhecendo as características intrínsecas dessas intoxicações, sobretudo nas regiões com maior crescimento desses agravos^(24)^.

### Limitações do estudo

O presente estudo apresentou como limitação o direcionamento temático somente para a visão dos enfermeiros. Espera-se que este estudo se torne referência para pesquisas que abordem a temática acerca das subnotificações de intoxicação exógena secundária pelo uso de agrotóxicos no ambiente rural, a partir dessa perspectiva dos profissionais, trazendo a possibilidade de um olhar por meio de outros pontos de vista.

### Contribuições para a área da Enfermagem, Saúde ou Política Pública

O estudo contribui com informações relevantes acerca das subnotificações de intoxicação exógena entre os trabalhadores do ambiente rural que, muitas vezes, são prejudiciais para os sistemas de informação em saúde. Nesse sentido, as estratégias elencadas pelos profissionais permitem uma reflexão-ação sobre o que é feito atualmente e como esse processo pode ser melhorado, com a finalidade de reduzir subnotificações e, consequentemente, direcionar políticas públicas de conscientização para a população rural sobre os riscos do manuseio incorreto ou equivocado de sustâncias, como os agrotóxicos. Além disso, o enfermeiro como agente da linha de frente, atuante em unidades de atenção primária e secundária, depara-se com a situação imediata, portanto, torna-se fundamental saber reconhecer os sinais e sintomas, realizar uma avaliação minuciosa, pautada na individualidade do paciente, para o correto diagnóstico e, posteriormente, notificação adequada.

## CONSIDERAÇÕES FINAIS

As estratégias utilizadas por enfermeiros acerca das subnotificações por uso de agrotóxicos no meio rural são importante ferramenta para a saúde pública. Os enfermeiros, como profissionais que atuam diretamente na assistência à saúde dos trabalhadores rurais, desempenham um papel importante na realização da notificação de acidentes de trabalho pelo uso de agrotóxicos. Para melhorar esse processo, precisam implementar estratégias efetivas, como manter-se atualizado, participando de programas de educação continuada e permanente acerca da temática; realizar a capacitação dos trabalhadores rurais que utilizam essas substâncias no seu trabalho, de forma que compreendam a importância da notificação de acidentes; realizar a busca ativa destes nos seus locais de trabalho e a notificação em fichas informatizadas, em um sistema integrado com as informações do paciente, facilitando o processo e contribuindo com a realização de novas pesquisas, garantindo conhecimento atualizado.

A notificação correta dos casos pode fortalecer as políticas de saúde e melhorar a segurança do trabalhador no meio rural. Torna-se necessário promover ações educativas e capacitações específicas para esses profissionais, visando aumentar sua percepção sobre os riscos relacionados ao uso de agrotóxicos e sua responsabilidade na notificação e prevenção desses acidentes. Além disso, a parceria entre enfermeiros e trabalhadores rurais apresenta-se como estratégia efetiva na prevenção e controle das intoxicações exógenas. A troca de conhecimentos contribui para melhorar a vida dos trabalhadores expostos ao uso de agrotóxicos, bem como tem potencial para reduzir os custos sociais e econômicos associados a esses acidentes.
